# Classification of Thyroid Follicular Lesions Based on Nuclear Texture Features—Lesion Size Matters

**DOI:** 10.1002/cyto.a.20982

**Published:** 2010-10-22

**Authors:** Konradin Metze, Rita C Ferreira, Randall L Adam

**Affiliations:** Department of Pathology, Faculty of Medical Sciences, University of CampinasBR 13081-970, Campinas, São Paulo, Brazil; Insitute of Computing, University of CampinasBR 13081-970, Campinas, São Paulo, Brazil

In their interesting contribution, Wang et al. (1) described an automated system for the diagnosis of follicular thyroid lesions, which was based on the analysis of nuclear texture features in histologic slides. According to the authors, the method was able to classify with 100% accuracy between normal thyroid tissue, follicular adenomas, and follicular carcinomas. However, the system had been trained with digitalized images of only few neoplasias, five follicular adenomas (mean lesion size of 4.8 cm), and five follicular carcinomas (a mean lesion size of 2.8 cm) as well as with images of nontumoral tissue.

Recently, it had been demonstrated that in follicular adenomas the chromatin texture was size dependent, when analyzing digitalized, gray-value transformed images of nuclei in routinely hematoxylin-eosin (HE)-stained paraffin sections. In nuclei of larger adenomas, the entropy values, the fractal dimension, and the diagonal moment were significantly increased, whereas the values of the second angular moment and local homogeneity were decreased, when compared with the nuclei of smaller adenomas (2). Therefore, we would like to ask whether the considerable size differences between follicular adenomas and carcinomas in the study of Wang et al. (1) might have contributed to the extraordinary classification success claimed by the authors. To test our hypothesis, we made the following simulation study based on adenomas and carcinomas from previous studies on thyroid neoplasias in our laboratory (2,3,4).

From each tumor, digitalized images of 100 randomly selected nuclei had been captured in bitmap format from routinely HE-stained 5-μm thick histologic sections (2,3). The nuclei were interactively segmented and then converted to an eight-bit gray scale by calculating the luminance. The material comprised 18 follicular adenomas and 24 microinvasive follicular carcinomas. The final diagnosis had always been done by a meticulous examination of serial step sections.

From this collection, we choose a combination of eight folicular carcinomas, so that their mean diameter of 2.78 cm had approximated well with that of the carcinomas from the study of Wang and coworkers (2.8 cm) (1). Then we selected with the help of random numbers, 100 sets of eight follicular adenomas each. The mean lesion size of these random sets varied between 1.98 and 5.21 cm.

**Figure 1:**
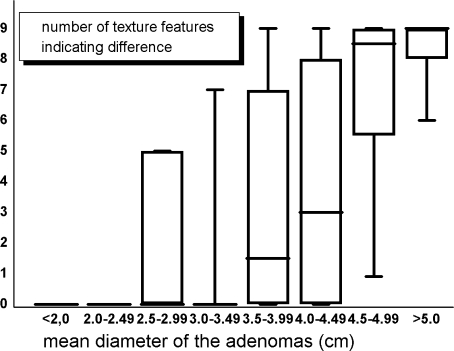
Box and Whiskers diagram showing relationship between mean lesion diameter of the adenoma group and the number of nuclear texture features separating significantly adenomas and carcinomas. Nine texture variables had been tested in each case. The number of those revealing significant differences in a *t*-test (*P* < 0.05) was plotted on the *y*-axis. With increasing size of the adenomas the chromatin architecture of adenomas is more different to that of carcinomas (Spearman's rank correlation coefficient *r* = 0.72; *P* < 0.0001).

If our previously mentioned hypothesis was correct, then the ability to discriminate between nuclei of adenomas and carcinomas should vary with changing diameters of the adenoma cases. We calculated nuclear texture features using variables of the gray-level co-occurrence matrix (second angular moment, entropy, energy, peak prominence, standard deviation, cluster shade, and cluster prominence) as well as Shannon's entropy of the gray level histogram and the fractal dimension according to Sarkar. All these variables had shown to be useful for texture analysis in previous studies (1,4–10). In a further step, we created 100 pairs composed of (a) always the aforementioned group of eight carcinomas with a mean diameter of 2.78 cm and (b) one of the 100 randomly choosen adenoma sets with, of course, varying mean lesion size (see Supporting Information). For each pair, we compared the nine texture features with the help of the *t*-test (*P* < 0.05 for significant differences) and recorded, for each comparison, the percentage of statistically significant texture features by plotting these data versus the mean diameter of the adenoma set in a diagram (Fig. 1).

As we can easily notice, in very large adenomas nearly all nuclear texture features were significantly different from those of the carcinoma group. But these differences were rapidly vanishing, when we compared the carcinomas with smaller adenomas. With a mean adenoma lesion size of 4.8 cm, as in the study under discussion (1), approximately 80% of the texture parameters showed significant differences. However, when the mean diameters of adenomas and carcinomas were very similar (∼2.8 cm), this number had dropped below 20%. There was a highly significant direct correlation (Spearman's correlation coefficient *r* = 0.72; *P* < 0.0001) between the mean lesion size of the adenoma cases and the percentage of significant different texture features between adenomas and carcinomas.

We believe that the extraordinary good discrimination results reported in the study of Wang et al. (1) might largely be based on this effect and would have been worse, if smaller adenomas had been included in the investigation. Therefore, the data presented in the study of Wang et al. (1) are not able to test the quality of the program. To get a realistic view of the diagnostic power of the proposed automated system, the authors should repeat the evaluation with a considerably larger data set, after increasing the number of adenomas and carcinomas, both comprising a large spectrum of tumor diameters.
