# Supplementary material for: Classification of Thyroid Follicular Lesions Based on Nuclear Texture Features—Lesion Size Matters
Source: Cytometry A. 2010 Oct 22;77A(12):1101–2. doi: 10.1002/cyto.a.20982 (PMC3051835; doi:10.1002/cyto.a.20982)
Supplement: Supplementary file 1 [file cyto077A-1101-SD1.doc]

Supplementary file Communication to the Editor Metze et al 2010

Data used for the simulation study

Arquivo diameter diagnosis SecAngMon Entropy

1233990000.BMP 1.00 adenoma 0.003711 8.761637

7738970000.BMP 1.20 adenoma 0.004681 8.355244

8419980000.BMP 1.20 adenoma 0.002833 8.993793

0965020000.BMP 2.00 adenoma 0.003081 8.995770

1025090000.BMP 2.00 adenoma 0.002188 9.432309

4029010000.BMP 2.20 adenoma 0.004547 8.546668

1090700000.BMP 3.00 adenoma 0.002571 9.178424

8007980000.BMP 3.20 adenoma 0.003883 8.721070

1237400000.BMP 3.30 adenoma 0.003622 8.653413

1794010000.BMP 3.50 adenoma 0.003444 8.836887

2120020000.BMP 3.50 adenoma 0.002455 9.279970

1028690000.BMP 4.00 adenoma 0.001705 9.769408

6762020000.BMP 4.50 adenoma 0.002308 9.358165

3776010000.BMP 5.00 adenoma 0.002536 9.221190

3980010000.BMP 5.20 adenoma 0.002425 9.269728

1896010000.BMP 5.50 adenoma 0.003541 8.796734

1020970000.BMP 7.00 adenoma 0.001649 9.711356

8793980000.BMP 7.00 adenoma 0.001606 9.786065

1165000000.BMP 2.00 carcinoma 0.003268 8.944770

2759000000.BMP 2.20 carcinoma 0.002626 9.171124

6964990000.BMP 2.20 carcinoma 0.003285 8.925611

1220790000.BMP 2.30 carcinoma 0.003004 9.050948

1163000000.BMP 2.50 carcinoma 0.002922 8.979130

3187980000.BMP 3.20 carcinoma 0.003359 8.903040

0351980000.BMP 3.80 carcinoma 0.003046 8.947975

1700980000.BMP 4.00 carcinoma 0.003467 8.794520

Arquivo Energy PeakPr StandDev

1233990000.BMP 5761 0.0128 0.0009

7738970000.BMP 4323 0.0146 0.0010

8419980000.BMP 6544 0.0083 0.0006

0965020000.BMP 7576 0.0095 0.0006

1025090000.BMP 8990 0.0074 0.0006

4029010000.BMP 5259 0.0158 0.0010

1090700000.BMP 7070 0.0088 0.0007

8007980000.BMP 5748 0.0134 0.0010

1237400000.BMP 5237 0.0100 0.0007

1794010000.BMP 7046 0.0108 0.0007

2120020000.BMP 8073 0.0083 0.0006

1028690000.BMP 11064 0.0057 0.0005

6762020000.BMP 8139 0.0078 0.0006

3776010000.BMP 7202 0.0092 0.0008

3980010000.BMP 8584 0.0076 0.0005

1896010000.BMP 6100 0.0110 0.0008

1020970000.BMP 9327 0.0058 0.0005

8793980000.BMP 10417 0.0053 0.0005

Arquivo Energy PeakPr StandDev

1165000000.BMP 6952 0.0108 0.0008

2759000000.BMP 7327 0.0090 0.0007

6964990000.BMP 6231 0.0117 0.0008

1220790000.BMP 7260 0.0103 0.0007

1163000000.BMP 6558 0.0097 0.0007

3187980000.BMP 6825 0.0109 0.0008

0351980000.BMP 6392 0.0101 0.0008

1700980000.BMP 6373 0.0109 0.0007

Arquivo ClshadeClProm ShanE FDSarkar

1233990000.BMP 13893 1725097 3.8949 2.0248

7738970000.BMP 6536 701819 3.6814 2.0197

8419980000.BMP 3596 2154869 3.9664 2.0367

0965020000.BMP 13790 2995008 3.9249 2.0423

1025090000.BMP 9780 2435765 4.0299 2.0366

4029010000.BMP 14198 1423868 3.7787 2.0234

1090700000.BMP 12003 2444199 4.0088 2.0306

8007980000.BMP 15093 1651098 3.8389 2.0239

1237400000.BMP 2609 779638 3.7365 2.0314

1794010000.BMP 11073 1578339 3.9022 2.0294

2120020000.BMP 12791 3022991 4.0338 2.0345

1028690000.BMP 45444 1.04630e+07 4.2250 2.0441

6762020000.BMP 14176 3643668 4.0595 2.0330

3776010000.BMP 14696 2746575 4.0315 2.0286

3980010000.BMP 25012 5834428 4.0375 2.0406

1896010000.BMP 7164 1528141 3.8449 2.0275

1020970000.BMP 17533 4252361 4.1349 2.0371

8793980000.BMP 24816 7260769 4.2028 2.0451

1165000000.BMP 9037 1551092 3.8889 2.0312

2759000000.BMP 15795 2535580 4.0203 2.0281

6964990000.BMP 10150 1806407 3.8887 2.0298

1220790000.BMP 11535 2108871 3.9200 2.0309

1163000000.BMP 5275 1218775 3.8629 2.0306

3187980000.BMP 12021 1740016 3.9140 2.0278

0351980000.BMP 3212 1179206 3.8591 2.0256

1700980000.BMP 4952 1077173 3.8204 2.0279
